# Case Report: breast cancer presenting as dermatomyositis

**DOI:** 10.3389/fonc.2026.1907254

**Published:** 2026-09-18

**Authors:** Qinchuan Li, Lai Song, Yueping Chen, Ya Zou, Ke Li

**Affiliations:** 1 Department of Clinical Pharmacology, West China School of Medicine, Sichuan University, Sichuan University affiliated Chengdu Second People’s Hospital, Chengdu Second People’s Hospital, Chengdu, China; 2 Department of Oncology, West China School of Medicine, Sichuan University, Sichuan University affiliated Chengdu Second People’s Hospital, Chengdu Second People’s Hospital, Chengdu, China

**Keywords:** anti-TIF1-γ antibody, breast cancer, cancer-associated dermatomyositis, dermatomyositis, paraneoplastic syndrome, treatment resistance

## Abstract

**Background:**

Cancer-associated dermatomyositis (CADM) is a rare paraneoplastic syndrome characterized by inflammatory myopathy and distinctive cutaneous manifestations. Early recognition of an underlying malignancy is essential for improving patient outcomes.

**Case presentation:**

We report the case of a 48-year-old woman who initially presented with dermatomyositis as the first manifestation of breast cancer. She was admitted with progressive dysphagia, violaceous edematous erythema involving the face and neck, and proximal muscle weakness of the extremities. The patient had previously been diagnosed with idiopathic dermatomyositis at another institution and treated with glucocorticoids; however, her symptoms showed no significant improvement. Further evaluation at our hospital revealed markedly elevated muscle enzyme levels. Autoantibody testing demonstrated weak positivity for anti-Jo-1 antibodies and positivity for anti-Ro-52 antibodies. Both skin and muscle biopsies supported the diagnosis of dermatomyositis. Comprehensive malignancy screening subsequently identified invasive carcinoma of the right breast with axillary lymph node metastasis. Following treatment with glucocorticoids combined with two cycles of neoadjuvant chemotherapy consisting of paclitaxel and platinum-based agents, the patient’s skin lesions markedly regressed, muscle strength improved, and dysphagia was significantly alleviated. She subsequently underwent radical breast surgery followed by adjuvant radiotherapy, and her condition has remained stable during follow-up.

**Conclusion:**

A review of the literature indicates that age >40 years, dysphagia, rapid disease progression, and positivity for anti-transcription intermediary factor 1-gamma a*nti*-*TIF*1-*γ* antibodies are important risk factors for CADM. Thorough malignancy screening should be performed in all newly diagnosed patients with dermatomyositis, particularly for common associated malignancies such as breast cancer, lung cancer, ovarian cancer, and nasopharyngeal carcinoma. Clinicians should maintain a high index of suspicion for occult malignancy in patients who respond poorly to conventional dermatomyositis therapy. Early identification and effective treatment of the underlying malignancy are critical for improving the prognosis of CADM.

## Introduction

Dermatomyositis (DM) is a systemic autoimmune disorder characterized by distinctive skin manifestations and inflammatory muscle involvement, and is recognized as a major subtype within the spectrum of idiopathic inflammatory myopathies (IIMs) (1, 2). Numerous epidemiological studies have confirmed that patients with dermatomyositis (DM) have a significantly higher risk of developing malignancies than the general population, with approximately 15%–30% of patients developing a concurrent malignancy, a condition termed cancer-associated dermatomyositis (CADM) (3). Cancer-associated dermatomyositis (CADM) is an uncommon paraneoplastic syndrome whose clinical activity is frequently correlated with the presence of an underlying malignancy. The majority of associated cancers are identified within 3 years before or after the diagnosis of dermatomyositis (DM) (4–6). The pathogenesis may be related to the expression of shared antigens by tumor cells and the subsequent induction of cross-reactive immune responses (7, 8). The myositis-specific antibodies discovered in recent years, especially the anti-TIF1-γ antibody, have provided an important basis for risk assessment in CADM (9, 10). Among female patients, breast cancer is one of the most common associated solid tumors in CADM (11, 12). However, cases presenting with dysphagia and characteristic skin rashes as the initial manifestations and ultimately diagnosed with breast cancer remain relatively rare. It is noteworthy that some patients with CADM have a poor response to conventional doses of glucocorticoids and immunosuppressants, and this clinical feature may serve as an important clue to an underlying malignancy (13). Here we report a case of breast cancer initially presenting as dermatomyositis and review the relevant literature to explore its clinical characteristics, diagnostic approach, and treatment strategies, with the aim of improving clinicians’ understanding of CADM.

## Case report

### General information

A 48-year-old female patient was admitted in August 2020 with “progressive dysphagia for 3 months, and facial and neck erythema with muscle soreness for 2 months.”

Three months prior to admission, the patient developed dysphagia to solid foods without obvious cause, which she initially ignored. Two months prior, she gradually developed large patches of erythema on the face and neck, accompanied by swelling and itching, along with proximal muscle weakness of the limbs, muscle soreness, and weakness in the waist and abdomen. Subsequently, the rash extended to the abdomen and bilateral lateral thighs. Dysphagia progressively worsened, and she developed choking on drinking water. She had no significant past medical history and no family history of tumors.

The patient denied any joint pain or swelling, Raynaud’s phenomenon (cold-induced color changes of the digits), cough, shortness of breath, fever, night sweats, or unexplained weight loss. Chest CT performed at admission showed no evidence of interstitial lung disease (ILD).

### Diagnosis and treatment at another hospital

At the early stage of the disease, the patient consulted a dermatology department at another hospital, where she was clinically diagnosed with “idiopathic dermatomyositis” and received oral prednisone (40 mg/day). After treatment, the rash and muscle weakness showed only transient and mild improvement, but symptoms recurred and progressively worsened after two weeks, with persistent deterioration of dysphagia. Due to poor response to conventional glucocorticoid therapy, she was referred to our hospital for further diagnosis and treatment.

### Physical examination

Dermatological examination revealed purplish-red edematous erythema around both eyelids (Heliotrope sign), symmetric erythema in the V-area of the anterior neck and chest (V-sign), and shawl-like erythema on the posterior neck and shoulders (Shawl sign). Gottron papules were observed over the dorsum of the metacarpophalangeal and proximal interphalangeal joints bilaterally. Periungual telangiectasias were prominent. All of the above are characteristic cutaneous manifestations of dermatomyositis (14–16).

Breast examination revealed a firm mass measuring approximately 2 cm × 3 cm in the upper outer quadrant of the right breast, with ill-defined borders and poor mobility. Enlarged lymph nodes were palpable in the right axilla.

Neuromuscular examination showed proximal muscle strength grade IV/V in all four limbs, normal distal muscle strength, and positive muscle tenderness.

### Auxiliary examinations

Laboratory findings on admission are summarized in **Table 1** (blood biochemistry and myocardial enzymes) and **Table 2** (autoantibodies and tumor markers).

**Table 1:** Blood biochemistry and myocardial enzyme profile.

Parameter	Result	Reference range	Unit
CK	1567.5	24–195	U/L
AST	108	13–35	U/L
LDH	359	120–250	U/L
HBDH	377	72–182	U/L
hs-cTnT	43.0	0–14	ng/L
CK-MB	28.5	0–5	ng/mL
ESR	30	0–20	mm/h
Serum potassium (lowest)	2.74	3.5–5.5	mmol/L

**Table 2:** Autoantibodies and tumor-related markers.

Parameter	Result	Reference range	Note
ANA (nuclear granular pattern)	1:160	<1:100	Positive
Anti-Jo-1 antibody	Weakly positive	Negative	Suspicious
Anti-Ro-52 antibody	Positive	Negative	Positive
Ferritin	323.8	13–150	ng/mL
NSE	22.0	0–16.3	μg/L

### Pathological examination

#### Neck skin biopsy

The biopsy showed epidermal hyperkeratosis with focal atrophy, vacuolar degeneration of basal keratinocytes (a characteristic finding of DM), superficial dermal edema, and perivascular lymphocytic infiltration. Pigment incontinence and focal mucin deposition were also observed, findings consistent with the histopathological features of dermatomyositis (15, 16).

#### Left deltoid muscle biopsy

The biopsy demonstrated degeneration and necrosis of muscle fibers, perifascicular atrophy (a hallmark of DM), perimysial inflammation, interstitial myxoid change, and proliferation of small vessels accompanied by lymphocytic infiltration, consistent with inflammatory myopathy. The histopathological findings of the skin and muscle biopsies are shown in **Figure 1**.

**Figure 1:**
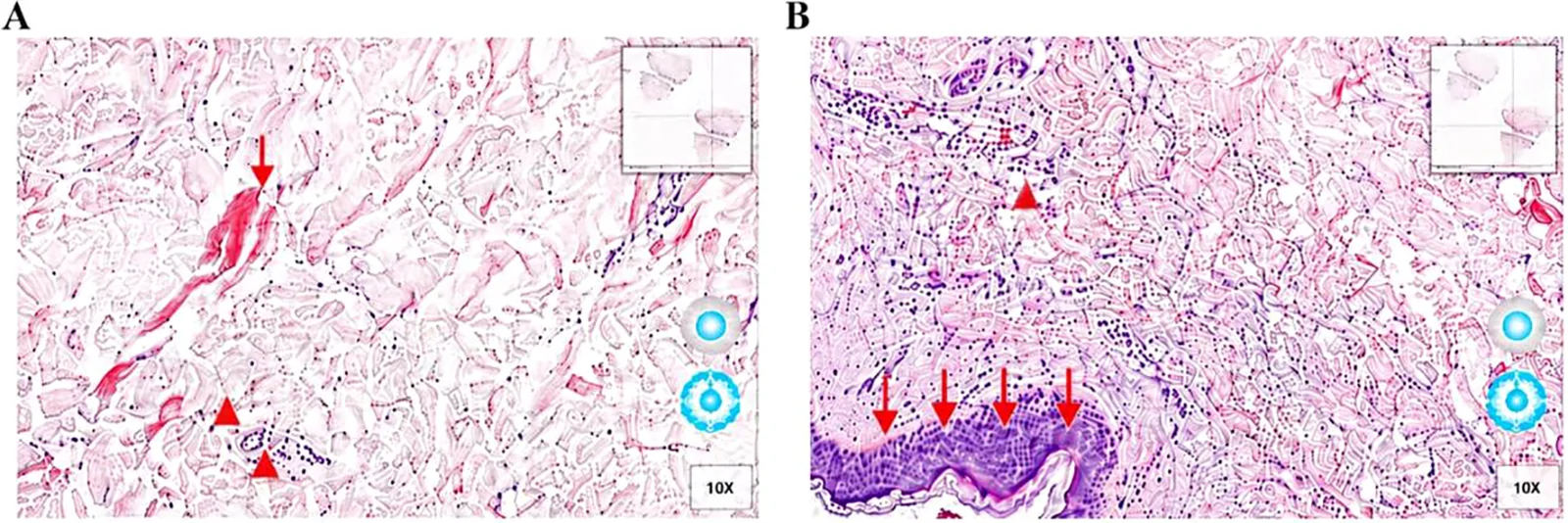
Histopathological examination of muscle and skin biopsies in a patient with cancer-associated dermatomyositis. Representative H&E staining images of muscle **(A)** and skin **(B)** biopsies. Muscle biopsy revealed perifascicular atrophy (arrows) accompanied by inflammatory infiltration in the perimysial regions (arrowheads). Skin biopsy demonstrated interface dermatitis characterized by vacuolar degeneration of basal keratinocytes (arrows) and perivascular lymphocytic infiltration (arrowheads). These pathological findings supported the diagnosis of dermatomyositis.

### Pathological examination of the breast lesion

Core needle biopsy of the right breast mass revealed invasive carcinoma. Istopathological examination showed atypical cells with pleomorphic nuclei, prominent nucleoli, and ductal formation, infiltrating into adjacent fibrous tissue. Histopathological examination of a right axillary lymph node biopsy demonstrated metastatic carcinoma. The breast biopsy and immunohistochemical features are presented in **Figure 2**.

**Figure 2:**
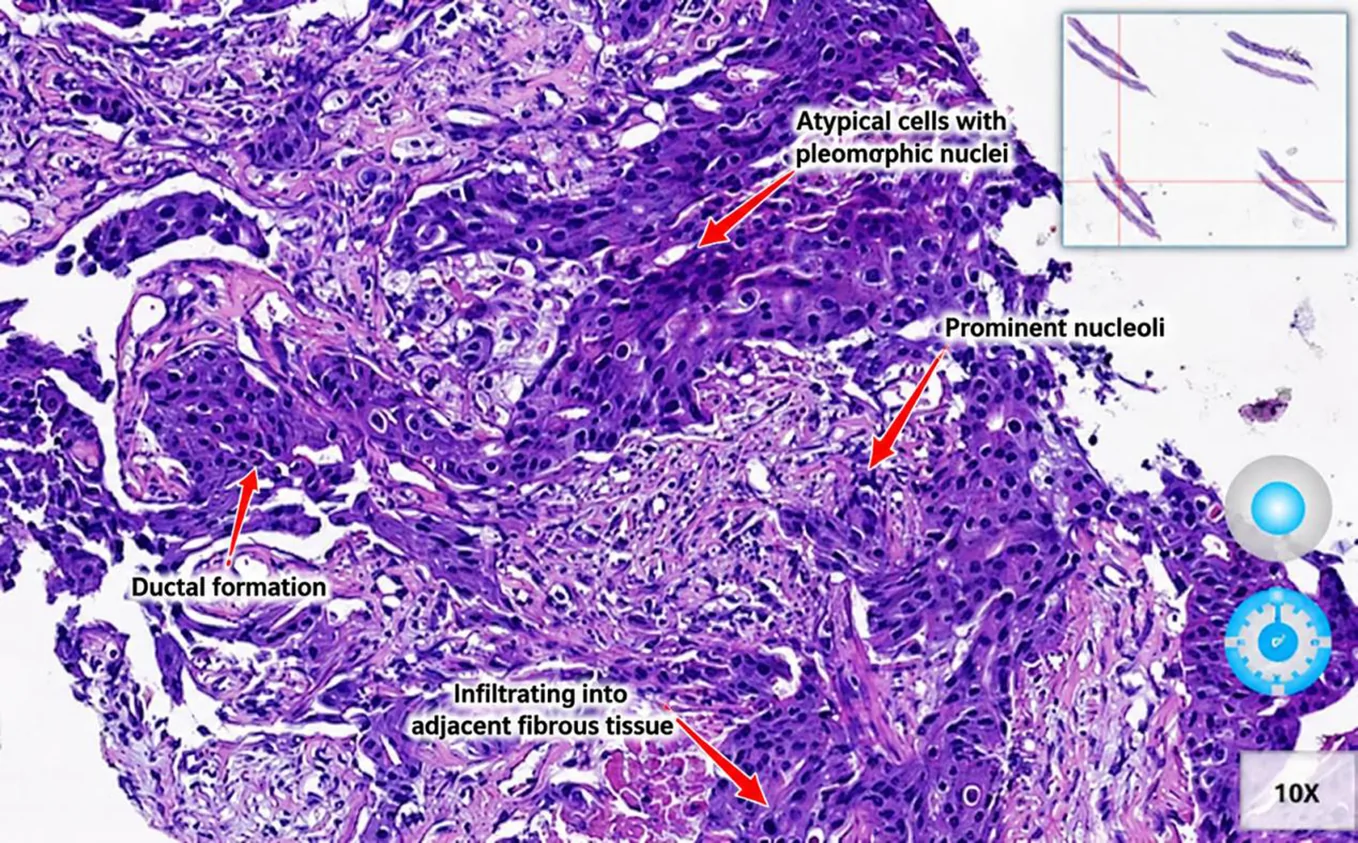
Histopathological examination of the breast lesion. H&E staining revealed invasive carcinoma cells arranged in irregular ductal structures and nests, with infiltration into adjacent fibrous tissue. Tumor cells showed pleomorphic nuclei, prominent nucleoli, and atypical cytological features. Arrows indicate ductal formation, pleomorphic nuclei, prominent nucleoli, and stromal invasion of carcinoma cells. Original magnification, ×10.

Immunohistochemical staining showed the following results: PCK (+), GATA3 (+), ER (−), PR (−), TTF-1 (−), P63 (−), and CA125 (−). These findings were consistent with metastatic carcinoma of breast origin.

### Diagnosis

Based on the patient’s clinical presentation, laboratory investigations, histopathological findings, and imaging studies, a final diagnosis of cancer-associated dermatomyositis (CADM) secondary to right invasive breast carcinoma with axillary lymph node metastasis (clinical stage cT2N1M0) was established. Differential diagnoses considered included idiopathic dermatomyositis and other inflammatory myopathies.

### Treatment and follow-up

After admission, the patient was treated with intravenous methylprednisolone sodium succinate at a dose of 80 mg/day, along with potassium supplementation and nutritional support. Given her poor response to previous glucocorticoid therapy and the confirmed diagnosis of breast cancer, a multidisciplinary team (MDT) discussion concluded that anti-tumor treatment should be prioritized (13, 17–19).The patient subsequently received two cycles of neoadjuvant chemotherapy with paclitaxel combined with a platinum-based agent. Following treatment, the violaceous erythema of the face and neck markedly regressed, proximal muscle strength of the extremities recovered to grade V, and both dysphagia and choking episodes while drinking water improved substantially.

The patient then underwent radical surgery for right breast cancer, followed by adjuvant radiotherapy. During follow-up, the symptoms of dermatomyositis remained in sustained remission without significant recurrence, and her overall condition remained stable. **Figure 3** presents the timeline of the diagnostic workup, treatment course, and clinical outcomes.

**Figure 3:**
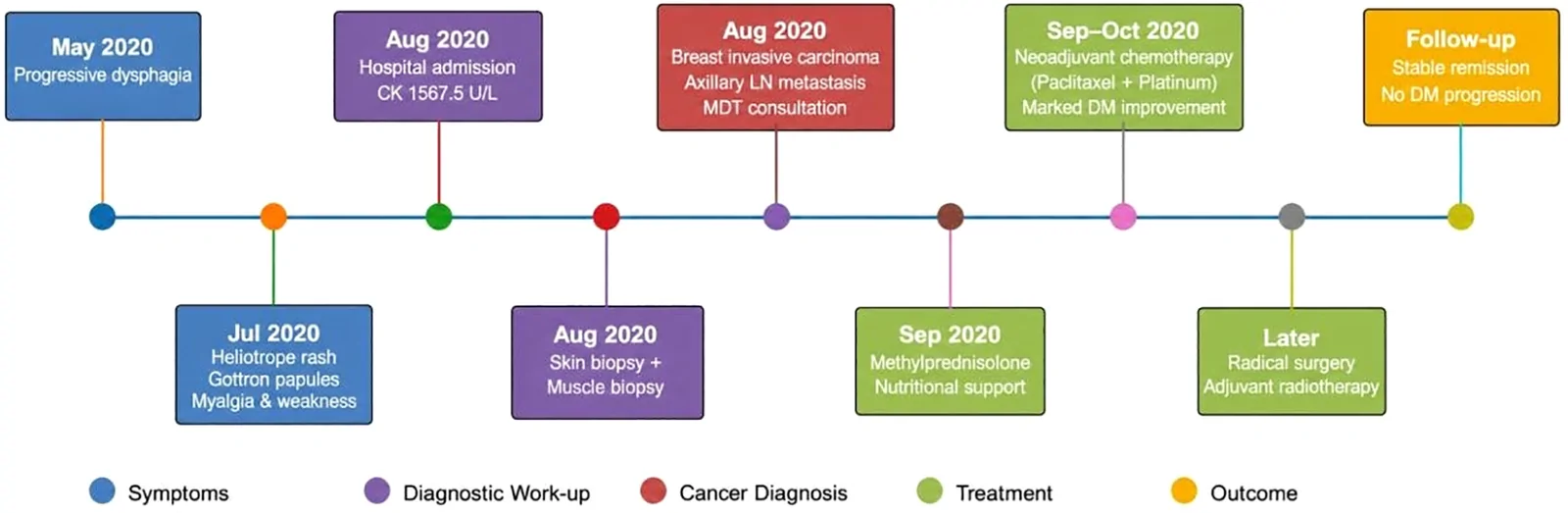
Presents the timeline of the diagnostic workup, treatment course, and clinical outcomes.

## Discussion

The association between dermatomyositis (DM) and malignancy has been well established (20). Previous studies have demonstrated that patients with DM have a 4- to 6-fold higher risk of developing malignancies compared with the general population, with the highest risk occurring within 3 years before or after the diagnosis of DM (21, 22). Among women, breast and ovarian cancers are the most frequently associated malignancies, whereas lung cancer, gastric cancer, and nasopharyngeal carcinoma are more commonly observed in men (23).

The present patient exhibited several high-risk features for cancer-associated dermatomyositis (CADM), including age greater than 40 years, acute disease onset, prominent dysphagia, and markedly elevated muscle enzyme levels. Notably, dysphagia has been identified in multiple studies as an important predictor of underlying malignancy in patients with DM (24). Therefore, the presence of dysphagia should prompt thorough malignancy screening.

An important feature of this case was the poor response to conventional glucocorticoid therapy. The patient initially received standard-dose prednisone for presumed idiopathic DM at another institution, resulting in only transient and minimal symptom relief, followed by rapid clinical deterioration. Such treatment resistance is not uncommon in CADM. Several studies have reported that patients with CADM generally respond less favorably to glucocorticoids and immunosuppressive agents than those with idiopathic DM (25). This phenomenon is thought to result from persistent tumor-driven immune dysregulation, which sustains disease activity despite immunosuppressive treatment. Consequently, when the underlying malignancy remains uncontrolled, immunosuppressive therapy alone is often insufficient. Clinicians should therefore maintain a high index of suspicion for CADM in patients whose symptoms fail to improve or continue to worsen after 2–4 weeks of standard treatment, and comprehensive cancer screening should be undertaken promptly.

In recent years, increasing attention has been directed toward the role of myositis-specific autoantibodies in assessing the risk of malignancy in DM. Among these, anti-transcription intermediary factor 1-gamma (anti-TIF1-γ) antibodies, also known as anti-p155 antibodies, are considered one of the strongest serological markers associated with cancer, and positivity is associated with a significantly increased risk of malignancy (26, 27). In addition, anti-nuclear matrix protein 2 (anti-NXP2) antibodies and anti-small ubiquitin-like modifier activating enzyme (anti-SAE) antibodies have also been linked to an elevated cancer risk (26). Emerging evidence suggests that increased serum levels of soluble programmed death-ligand 1 (sPD-L1) may likewise indicate an increased likelihood of underlying malignancy (28). Therefore, comprehensive testing for myositis-specific autoantibodies is recommended in newly diagnosed DM patients, particularly those older than 40 years, to facilitate risk stratification and guide malignancy screening strategies (27, 29, 30). The 2023 IMACS international guideline for IIM-associated cancer screening strongly recommends comprehensive myositis-specific autoantibody testing in all adult patients with new-onset IIM to assist cancer risk stratification, and identifies age >40 years, dysphagia, and anti-TIF1-γ positivity as high-risk factors, further supporting the clinical approach taken in this case (29).

It is important to note that in the present case, anti-Jo-1 antibodies were only weakly positive. Anti-Jo-1 antibodies are primarily associated with the antisynthetase syndrome—characterized by arthritis, Raynaud’s phenomenon, mechanic’s hands, and interstitial lung disease—rather than cancer-associated myositis. The absence of these clinical features in our patient, together with the weak anti-Jo-1 positivity, further suggests that the anti-Jo-1 result was unlikely to be the driver of her presentation. This underscores the clinical lesson that even in the absence of classic high-risk autoantibodies such as anti-TIF1-γ, the combination of treatment-resistant dermatomyositis, age >40 years, and dysphagia should prompt thorough malignancy screening.

The therapeutic approach to CADM differs from that of idiopathic DM. Although glucocorticoids and immunosuppressive agents may partially alleviate inflammation, their efficacy is often limited (17, 31). Numerous case reports and clinical studies have demonstrated that effective treatment of the primary malignancy is frequently accompanied by substantial improvement or complete remission of dermatomyositis symptoms, whereas tumor recurrence or progression is commonly associated with disease exacerbation (32–34).Therefore, aggressive treatment of the underlying malignancy should be regarded as the cornerstone of CADM management. In the present case, the patient’s cutaneous manifestations, muscle weakness, and dysphagia improved markedly after only two cycles of neoadjuvant chemotherapy, further supporting the concept that “controlling the tumor is key to controlling dermatomyositis.” This observation also highlights the limitations of glucocorticoid therapy alone in patients with tumor-driven disease.

### Strengths and limitations

The main strength of this case report is the detailed documentation of treatment resistance as a red flag for underlying malignancy, supported by a comprehensive literature review. A limitation is that anti-TIF1-γ antibody testing was not performed due to assay unavailability at the time of diagnosis.

## Patient perspective

The patient reported that her progressive dysphagia and skin rash severely affected her daily life and caused significant anxiety before the diagnosis was established. She expressed relief after the breast cancer was identified and a clear treatment plan was initiated. Regarding the neoadjuvant chemotherapy, she was initially concerned about potential side effects such as nausea, fatigue, and alopecia; however, she felt that the marked improvement in her skin and muscle symptoms outweighed these temporary inconveniences. She was satisfied with the treatment outcomes and continues to attend regular follow-up visits. She agreed to the publication of this case report to help other patients with similar presentations.

## Conclusion

Dermatomyositis may serve as the initial manifestation of breast cancer. Clinicians should maintain a high index of suspicion for underlying malignancy in patients with DM who are older than 40 years, present with dysphagia, exhibit rapid disease progression, or harbor high-risk myositis-specific autoantibodies. Importantly, a poor response to conventional dermatomyositis therapy or continued clinical deterioration after 2–4 weeks of standard treatment should be regarded as a warning sign of cancer-associated dermatomyositis (CADM), prompting timely and comprehensive malignancy screening. Standardized cancer screening, multidisciplinary team-based management, and a treatment strategy centered on effective control of the underlying malignancy are essential for improving outcomes in patients with CADM.

## Data Availability

The original contributions presented in the study are included in the article/supplementary material. Further inquiries can be directed to the corresponding authors.
